# Rechallenge With Anti-Human Epidermal Growth Factor Receptor 2 Therapy Guided by Liquid Biopsy in *HER2*-Amplified Metastatic Colorectal Cancer

**DOI:** 10.1200/PO-26-00515

**Published:** 2026-07-29

**Authors:** Patricia Garcia-Pastor, Javier Ros, Iosune Baraibar, Francesc Salva, Clara Salva, Ariadna Garcia, Caterina Vaghi, Josep Tabernero, Ana Vivancos, Elena Elez

**Affiliations:** ^1^ Medical Oncology Department, Vall d’Hebron University Hospital, Barcelona, Spain; ^2^ Vall d’Hebron Institute of Oncology (VHIO), Barcelona, Spain; ^3^ Medical Oncology Department, Niguarda Cancer Center, Grande Ospedale Metropolitano, Niguarda, Milan, Italy

## Background

Human epidermal growth factor receptor 2 (HER2, ERBB2) amplification or overexpression occurs in approximately 3%-6% of patients with metastatic colorectal cancer (mCRC), predominantly in *RAS*/*BRAF* wildtype tumors.^1,2^ Clinically, *HER2*-positive mCRC represents a distinct molecular subset enriched in left-sided primary tumors and often brain metastases.^3^

Dual HER2 blockade with trastuzumab in combination with pertuzumab, lapatinib, or tucatinib, as well as antibody-drug conjugates such as trastuzumab deruxtecan, have achieved objective response rates of approximately 30%-40% in prospective clinical trials.^4-6^ These results have established *HER2* amplification as a predictive biomarker of response to targeted therapies and have led to the incorporation of anti-HER2 agents into treatment algorithms for refractory disease.^7^ Rather than a binary variable, *HER2* amplification should be understood as a quantitative and context-dependent biomarker that shapes signaling dependency across the epidermal growth factor receptor (EGFR)-HER2 axis.^8,9^ This biologic continuum supports a nonmutually exclusive model of pathway activation paving the way for sequential or alternating therapeutic strategies targeting EGFR and HER2 in selected patients.

Despite these advances, eventually almost all patients will develop disease progression and resistance to HER2-targeted therapies reflecting the dynamic nature of tumor clonal evolution. Anti-HER2 rechallenge has been shown to be feasible, with response rates of around 20% in a retrospective series.^10^ However, robust selection criteria and predictive biomarkers of response remain lacking. In this context, circulating tumor DNA (ctDNA) analysis has emerged as a key tool for real-time, noninvasive assessment of tumor genomics and clonal evolution tracking, paving the way to rechallenge strategies in mCRC. The biological rationale for these strategies is based on the observation that cancer clones harboring mutations associated with resistance to mitogen-activated protein kinase (MAPK) inhibition emerge under the selective pressure of targeted therapy and often decay after treatment discontinuation, potentially restoring sensitivity to this therapeutic strategy.^11-13^ Beyond baseline molecular profiling, ctDNA enables longitudinal monitoring of *HER2* amplification levels and the identification of emergent resistance mechanisms, including alterations affecting MAPK signaling or loss of HER2 dependency.^9^ This dynamic perspective provides biologic rationale for treatment, sequencing, and rechallenge strategies, analogous to those established in anti-EGFR therapy. On the basis of this rationale, we present two patients with mCRC and *HER2* amplification who were treated with anti-HER2 therapy using liquid biopsy to guide the rechallenge strategy.^10^

## Methods

This is a retrospective case review of two patients with mCRC. Both patients underwent subsequent antiHER2 therapies, with concurrent longitudinal assessments of ctDNA. ctDNA was measured using the Guardant360 CDx next-generation sequencing (NGS) technology. Detected variants were reported as variant allele fraction, defined as the percentage of mutant alleles relative to total cell-free DNA at the specific locus. Imaging modalities included computed tomography scan, as clinically indicated. ctDNA testing was used off-label at the discretion of treating physicians. Patient characteristics were abstracted by chart review from Vall Hebron University Hospital, Barcelona, Spain. Both patients' relatives provided consent for publication of deidentified patient data.

**FIG 1.**
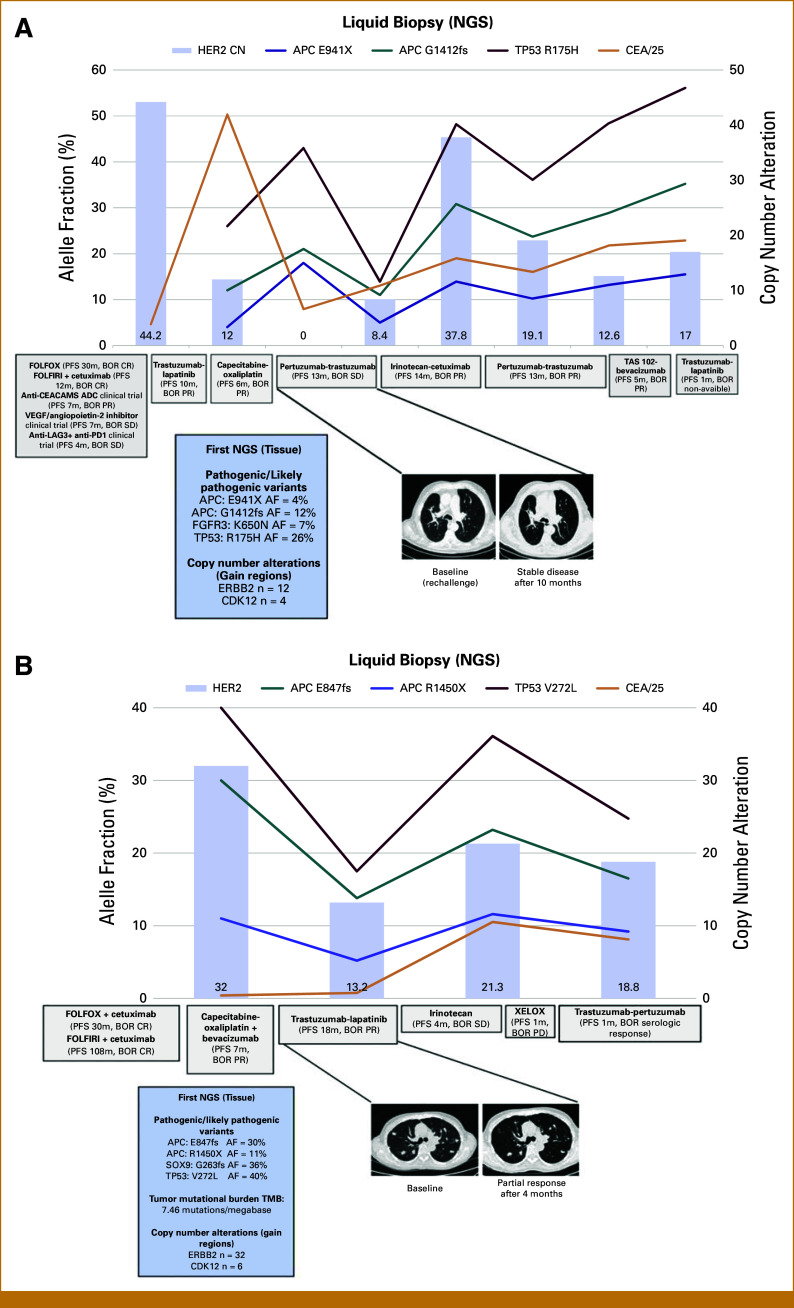
(A) Clinical evolution, PFS, key molecular findings, VAF, and CN variant evolution of patient 1. (B) Clinical evolution, PFS, key molecular findings, VAF, and CN variant evolution of patient 2. *HER2* amplification is represented using a bar chart on the secondary *y*-axis, CEA values are displayed as a scaled line plot (CEA/25) on the secondary *y*-axis, and allelic variants are expressed as percentage on the principal *y*-axis. AF, allele fraction; BOR, best overall response; CEA, carcinoembryonic antigen; CN, copy number; CR, complete disease; NGS, next-generation sequencing; PR, partial response; SD, stable disease; TMB, tumor mutational burden; VAF, variant allelic fraction.

Patient 1 was a 66-year-old man diagnosed with metastatic rectal adenocarcinoma, microsatellite stable (MSS), *RAS*/*BRAF* wildtype, with lung and liver spread. He received neoadjuvant chemoradiotherapy with folinic acid, fluorouracil, and oxaliplatin (FOLFOX), followed by surgery on the primary tumor and adjuvant FOLFOX with pulmonary partial response and hepatic complete response. Afterward, lung surgery was performed with adjuvant FOLFOX. Later, because of lung progression after 14 months, treatment with folinic acid, fluorouracil, and irinotecan (FOLFIRI) plus cetuximab was initiated with a progression-free survival (PFS) of 12 months. Subsequently, he was referred to our institution and was enrolled in several clinical trials, including an antibody-drug conjugate (antiCEACAM, PFS 7 months), novel anti-EGFR inhibitors (PFS, 7 months), novel antiangiogenic inhibitors (PFS, 7 months), and immune checkpoint inhibitors (antiPD1 plus an antiLAG3, PFS, 4 months). Upon progression, no tumor tissue was available; therefore, a plasma NGS was performed, which showed *HER2* amplification (copy number [CN], 44), leading to the initiation of treatment with trastuzumab-lapatinib (PFS, 10 months) with partial response as the best response. Subsequently, treatment with oxaliplatin and capecitabine was administered (PFS, 6 months), and upon progression, a new liquid biopsy revealed persistence of *HER2* amplification (CN, 12) with no genomic mechanisms of resistance, so pertuzumab plus trastuzumab was initiated with a long-lasting response (PFS, 13 months). At recurrence, plasma NGS was repeated, revealing lack of *HER2* expression; therefore, irinotecan plus cetuximab was administered with a long-lasting response (PFS, 14 months). Upon progression, subsequent liquid biopsy revealed *HER2* amplification (CN, 37.8), leading to trastuzumab plus pertuzumab rechallenge with disease stabilization (PFS 5 months), showing at the moment of progression an *HER2* CN decay (to 12). Afterward, he received trifluridine-tipiracil-bevacizumab (PFS 5 months). A subsequent liquid biopsy demonstrated mild increase of *HER2* amplification (CN, 17), whereupon rechallenge with trastuzumab plus lapatinib was started but after 1 month, the patient died because of disease progression (Fig 1A).

Patient 2 was a 53-year-old woman who was diagnosed with a left-sided, *RAS/BRAF* wildtype, MSS colon adenocarcinoma with peritoneal and ovarian spread. She received FOLFOX plus cetuximab and FOLFIRI plus cetuximab, with a long-lasting response and complete response with both regimens, followed by capecitabine-oxaliplatin plus bevacizumab (PFS, 7 months) after progression to anti-EGFR therapy. When progression occurred, the patient was referred to our institution, where a liquid biopsy revealed *HER2* amplification (CN, 32). The patient started treatment with trastuzumab-lapatinib achieving stable disease lasting for 18 months. Upon progression, *HER2* CN decayed to 12, then treatment with irinotecan monotherapy was initiated with poor response (PFS, 4 months), followed by capecitabine-oxaliplatin (PFS, 1 month). A new liquid biopsy was performed, revealing *HER2* CN increase (21.3) without an acquired genomic mechanism of resistance. Therefore, trastuzumab and pertuzumab were started, and despite clinical and tumor marker improvement, the patient died because of lung infection before radiologic assessment was performed (Fig 1B).

## Discussion

*HER2* amplification is a well-established predictive biomarker of response to anti-HER2 therapies in mCRC, while also representing a negative predictor of response to anti-EGFR agents.^14^ Tumors with high *HER2* CN are consistently associated with lack of benefit from anti-EGFR therapies, whereas those with lower levels of amplification may retain partial EGFR dependence.^9,15^ Conversely, emerging retrospective data support a gene-dosage–driven efficacy gradient for anti-HER2 therapies, although a consensus cutoff has not yet been established.^16^

Despite the advances, resistance to anti-HER2 therapies eventually emerges, highlighting the need for adaptive and precision-guided strategies. Beyond its role as a prognostic and predictive biomarker and surrogate of tumor burden, ctDNA enables real-time monitoring of clonal evolution and may inform rechallenge approaches. This paradigm has been well established in *RAS*/*BRAF* wildtype mCRC treated with anti-EGFR therapy, where resistant clones driven by MAPK pathway alterations emerge under treatment pressure but decay after drug withdrawal, restoring sensitivity and enabling molecularly guided rechallenge.^11^ A similar biologic rationale may apply to HER2-amplified tumors, in which resistance mechanisms, such as acquisition of MAPK alterations or loss of HER2 expression, are increasingly recognized and can also be dynamic and reversible.^17^ Therefore, longitudinal ctDNA assessment may help identify patients suitable for anti-HER2 rechallenge.^10^

These patient cases illustrate the dynamic nature of *HER2*-amplified mCRC and support ctDNA as a real-time biomarker that may help guide therapeutic sequencing. Liquid biopsy revealed temporal heterogeneity of HER2 status, with amplification emerging, disappearing, and re-emerging under different therapeutic pressures. These observations suggest that HER2-driven clones may decay after treatment discontinuation, mirroring dynamics reported in anti-EGFR rechallenge. This biologic behavior reinforces the concept that resistance to anti-HER2 therapy may be adaptive and reversible, thereby providing a biologic basis for rechallenge strategies in selected patients.

Patient 1 exemplifies the potential of this approach, where each progression on anti-HER2 therapy was associated with a decrease in *HER2* CN, followed by re-expansion before subsequent HER2-based rechallenge. This cyclical pattern suggests that alternating HER2-targeted and non‑HER2-targeted therapies may promote clonal reshaping and restore sensitivity. Moreover, the reduction in *HER2* CN preceded renewed sensitivity to anti-EGFR therapy, underscoring the reciprocal interplay between HER2 and EGFR signaling. By contrast, patient 2 experienced long-lasting clinical and radiologic benefit from initial anti-HER2 therapy. At progression, ctDNA demonstrated a reduction in *HER2* copy number, with a later increase after a subsequent chemotherapy regimen, supporting the biologic rationale for rechallenge with a decrease in tumor markers suggesting potential antitumor activity.

Collectively, these findings emphasize that *HER2* amplification in mCRC should be considered a dynamic biomarker rather than a static alteration and that liquid biopsy can accurately track changes in *HER2* copy number, thereby guiding treatment strategies. Importantly, *HER2* amplification should not be interpreted as an absolute resistance marker to anti-EGFR therapy, but rather as a context-dependent variable influenced by CN and clonal dynamics. Ultimately, precision oncology in mCRC will rely not only on identifying actionable alterations but also on understanding their temporal dynamics.^10^ In this setting, liquid biopsy-guided therapeutic sequencing represents a promising strategy to optimize outcomes in patients with *HER2*-amplified disease. Analogous to ctDNA-guided anti-EGFR rechallenge strategies in *RAS/BRAF* wildtype mCRC,^12^ longitudinal monitoring of HER2 status through liquid biopsy may represent a promising approach not only to refine patient selection for anti-HER2 rechallenge in mCRC but also to capture dynamic changes in HER2 status over time. This strategy could enable a more personalized treatment approach, guiding both the optimal timing of anti-HER2 re-treatment and the selection of the most appropriate HER2-targeted agent according to the evolving molecular profile of the disease.^9,18^
